# Recent advances in the therapeutic management of calcium pyrophosphate deposition disease

**DOI:** 10.3389/fmed.2024.1327715

**Published:** 2024-03-11

**Authors:** Paraskevi V. Voulgari, Aliki I. Venetsanopoulou, Alexandros A. Drosos

**Affiliations:** Department of Rheumatology, School of Health Sciences, Faculty of Medicine, University of Ioannina, Ioannina, Greece

**Keywords:** calcium pyrophosphate deposition disease, NSAIDs, glucocorticoids, colchicine, anakinra, hydroxychloroquine, methotrexate

## Abstract

Calcium pyrophosphate deposition (CPPD) disease is a form of crystal-induced arthropathy that arises from the accumulation of calcium pyrophosphate crystals within joints and soft tissues. This process leads to inflammation and damage to the affected joints. It can present asymptomatically or as acute or chronic inflammatory arthritis. Risk factors and comorbidities, including prior joint injury, osteoarthritis, hereditary or familial predisposition, and metabolic diseases, should be evaluated in CPPD cases. The management of CPPD remains a challenge in the sparsity of randomized controlled trials. The lack of such trials makes it difficult to establish evidence-based treatment protocols for CPPD. This review provides an overview of the current pharmacological management of CPPD, focusing on reducing inflammation, alleviating symptoms, and preventing acute flares. Non-steroidal anti-inflammatory drugs (NSAIDs), corticosteroids, and colchicine are effective in managing acute CPP arthritis. Colchicine may also be used prophylactically to prevent recurrent flares. In cases where other treatments have failed, anakinra, an interleukin-1 receptor antagonist, can be administered to alleviate acute flares. The management of chronic CPP inflammatory arthritis includes NSAIDs and/or colchicine, followed by hydroxychloroquine, low-dose glucocorticoids, and methotrexate, with limited data on efficacy. Tocilizumab can be used in refractory cases. In small studies, synovial destruction using intra-articular injection of yttrium 90 can decrease pain. To date, no disease-modifying therapies exist that reduce articular calcification in CPPD.

## 1 Introduction

Calcium pyrophosphate crystal deposition (CPPD) disease represents a prevalent form of crystal-induced arthropathy (1). It is hallmarked by calcium pyrophosphate (CPP) crystals accumulation in joints and soft tissues, leading to inflammation and damage. CPPD affects different joints, such as the knees, wrists, ankles, elbows, toes, shoulders, and hips (1, 2).

While CPPD may occur sporadically, understanding the associated risk factors and comorbidities, such as previous joint injury, metabolic disorders (e.g., primary hyperparathyroidism, hemochromatosis, hypophosphatasia, and hypomagnesemia), hereditary predisposition, and osteoarthritis (OA), is crucial (3–5). In younger patients with polyarthritis, it is crucial to consider metabolic or familial predisposition (3–5).

The cause of CPPD disease is not yet fully understood. However, the process starts with the formation of CPP crystals in the cartilage’s pericellular matrix (6, 7). Inorganic pyrophosphate, derived from extra-cellular Adenosine triphosphate (ATP), plays a crucial role in CPPD (7). Once formed, CPP crystals activate components of the NLRP3 inflammasome and create neutrophil extra-cellular traps, triggering inflammation. Additionally, CPP crystals exert direct catabolic effects on chondrocytes and synoviocytes, leading to the production of destructive matrix metalloproteinases and prostaglandins. Furthermore, CPP crystal deposits in articular cartilage can alter its mechanical properties, resulting in joint damage (6–8).

It is important to distinguish CPPD disease from chondrocalcinosis, which involves radiographic calcification in hyaline cartilage and/or fibrocartilage. The prevalence of articular chondrocalcinosis is around 15% of individuals aged 60 or older (9). However, the calcification detected by imaging or histological examination does not always indicate CPPD (1, 3, 4).

The management of CPPD requires a comprehensive approach to alleviate symptoms and control the underlying factors contributing to joint inflammation. This review analyzes recent advances in the therapeutic management of CPPD disease, addressing the challenges posed by its heterogeneous clinical presentations, the lack of a standardized treatment approach, and the limited availability of high-quality evidence.

## 2 Clinical phenotypes and diagnosis

The CPPD manifests in various clinical presentations, including asymptomatic CPPD, OA with CPPD, acute CPP crystal arthritis, and chronic CPP crystal inflammatory arthritis (1, 2).

Asymptomatic CPPD is a condition that lacks clinical manifestations, and it is identified incidentally during imaging for other reasons. When CPPD is found in a joint with changes of OA in imaging or histological examination, it is referred to as OA with CPPD. Acute CPP crystal arthritis is a self-limiting synovitis that has an acute onset and is associated with CPPD. It replaces the term “pseudogout.” Chronic CPP crystal arthritis, on the other hand, is a chronic inflammatory oligoarthritis or polyarthritis. It should be considered in the differential diagnosis of rheumatoid arthritis (RA) and other chronic inflammatory joint diseases in older adults (1–5). Severe joint degeneration in CPPD may resemble neuropathic arthropathy (pseudo-neuropathic joint), but it is instead characterized by normal neurological function (4). Spinal involvement leading to spinal stiffness, bony ankylosis, and syndromes of spinal cord or nerve compression may also occur, most commonly encountered in familial CPPD disease (5).

The diagnosis of CPPD is established when characteristic CPP crystals, which are weakly positive birefringent, mostly rhomboid or rod-shaped, are present in the synovial fluid or tissue from the affected joint (1). Radiographic chondrocalcinosis supports the diagnosis of CPPD, but its absence does not exclude it (1–3, 10).

**Figure 1:**
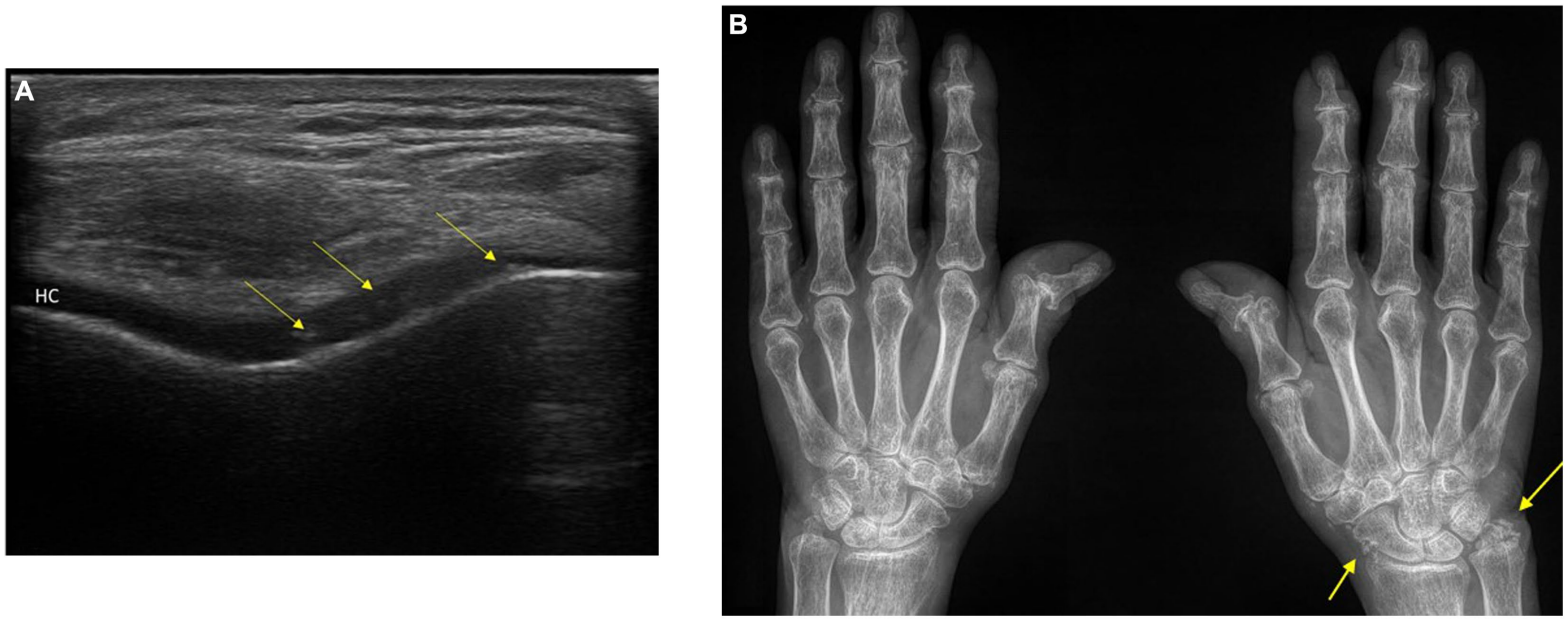
**(A)** Ultrasonography of the knee: typical appearance of calcium pyrophosphate deposition disease (CPPD) in the hyaline cartilage (HC) of the knee. **(B)**. Conventional radiograph of the hands: Chondrocalcinosis of the triangular fibrocartilage of the right wrist and periarticular tissues. Osteoarthritis of the first carpometacarpal and joint space narrowing in the DIP and PIP joints with erosions. The patient has findings of CPPD arthropathy and osteoarthritis. Arrows: calcium pyrophosphate (CPP) crystal deposits. DIP, distal interphalangeal; PIP, proximal interphalangeal; HC, anechoic (black) layer above the bone profile.

An international group of experts has recently established clear guidelines for identifying CPPD-related calcifications across different imaging techniques (11). Conventional radiography is considered the basic tool for detecting calcifications in fibrocartilage, hyaline cartilage, synovial membranes, joint capsules, and tendons. These calcifications appear as distinct linear or punctate opacities on conventional radiographs. Ultrasonography can demonstrate CPPD in peripheral joints, appearing as thin hyperechoic bands within hyaline cartilage and hyperechoic sparkling spots in fibrocartilage. Sensitivity and specificity appear excellent and possibly better than conventional x-rays (12) (Figures 1A,B). Conventional Computed tomography (CT) imaging further enhances the identification of CPPD-related calcifications (11) and can be useful in patients with atypical sites of CPPD such as the atlantooccipital joint or CPPD in and around atlantoaxial articulation (crowned dens syndrome) (5, 10). It reveals linear or punctate opacities with lower density (<300 Hounsfield Units). These calcifications are primarily located within fibrocartilage, hyaline articular cartilage, synovial membrane, joint capsule, and tendons.

Dual-energy CT (DECT) is an advanced imaging technique that can detect and identify CPP depositions in various anatomical structures. DECT reveals well-defined, linear, or punctate calcifications within fibrocartilage or hyaline cartilage. These calcifications appear thinner and less dense (<300 Hounsfield Units) compared to cortical bone. It can identify CPPD-related calcifications within the synovial membrane, joint capsule, or tendons exhibiting similar characteristics of well-defined, linear, or punctate calcifications with lower density (11).

The recently published 2023 American college of Rheumatology (ACR) /European League Against Rheumatism (EULAR) CCPD disease classification criteria have provided new guidelines for classifying patients presenting with joint pain, swelling, or tenderness whose symptoms are not fully explained by an alternative disease (13, 14). According to the criteria, presence of crowned dens syndrome or CPP crystals in synovial fluid is sufficient to classify a patient as having CPPD disease. Additionally, to classify a patient as having CCPD disease, a score of more than 56 points could be obtained through a set of weighted criteria that includes various factors such as clinical features, associated metabolic diseases, and the results of laboratory and imaging investigations (13).

## 3 CPPD management

The management of CPPD disease presents a challenge due to the sparsity of randomized controlled trials (RCTs) in this field (14–16). Thus, clinicians often rely on insights from the treatment of gout, another crystal deposition disease. The treatment options for CPPD aim to manage inflammation, alleviate the symptoms of acute and chronic diseases, and prevent acute flares. Notably, the recommendations outlined by EULAR in 2011 are primarily based on non-randomized trials, observational studies, and expert consensus rather than robust RCTs (14). Therefore, clinicians should approach the management of CPPD disease with an understanding of the lower evidence base and be cautious in applying treatment guidelines to individual patient cases.

Unlike gout, there is no proven method to eliminate CPP crystals (1). Asymptomatic chondrocalcinosis patients do not require treatment. However, patients with an underlying disorder associated with CPPD should receive therapy specific to the underlying disorder, although it usually does not reverse CPPD disease (1, 2, 14). Many agents used in CPPD management are derived from gout treatment, symptomatic OA, and RA. Recently, a systematic literature review was conducted to identify pharmacologic and non-pharmacologic treatment options for CPPD and describe their efficacy and safety (15). The review analyzed 22 studies that met the eligibility criteria, including 3 randomized double-blind controlled trials, 9 cohort studies, and 10 case series, involving 403 patients. The available high-quality evidence was limited. Furthermore, commonly administered agents such as non-steroidal anti-inflammatory drugs (NSAIDs), colchicine, and corticosteroids have not been evaluated by RCTs.

The primary management of CPPD involves managing inflammation and monitoring concomitant factors. However, sometimes surgical action may become necessary, especially when the adjacent soft tissue is damaged or if there is a symptomatic loss of cartilage or joint stability. In such situations, surgical procedures can help reduce pain and enhance joint mobility (17, 18).

## 4 Treatment of CPPD

**Figure 2:**
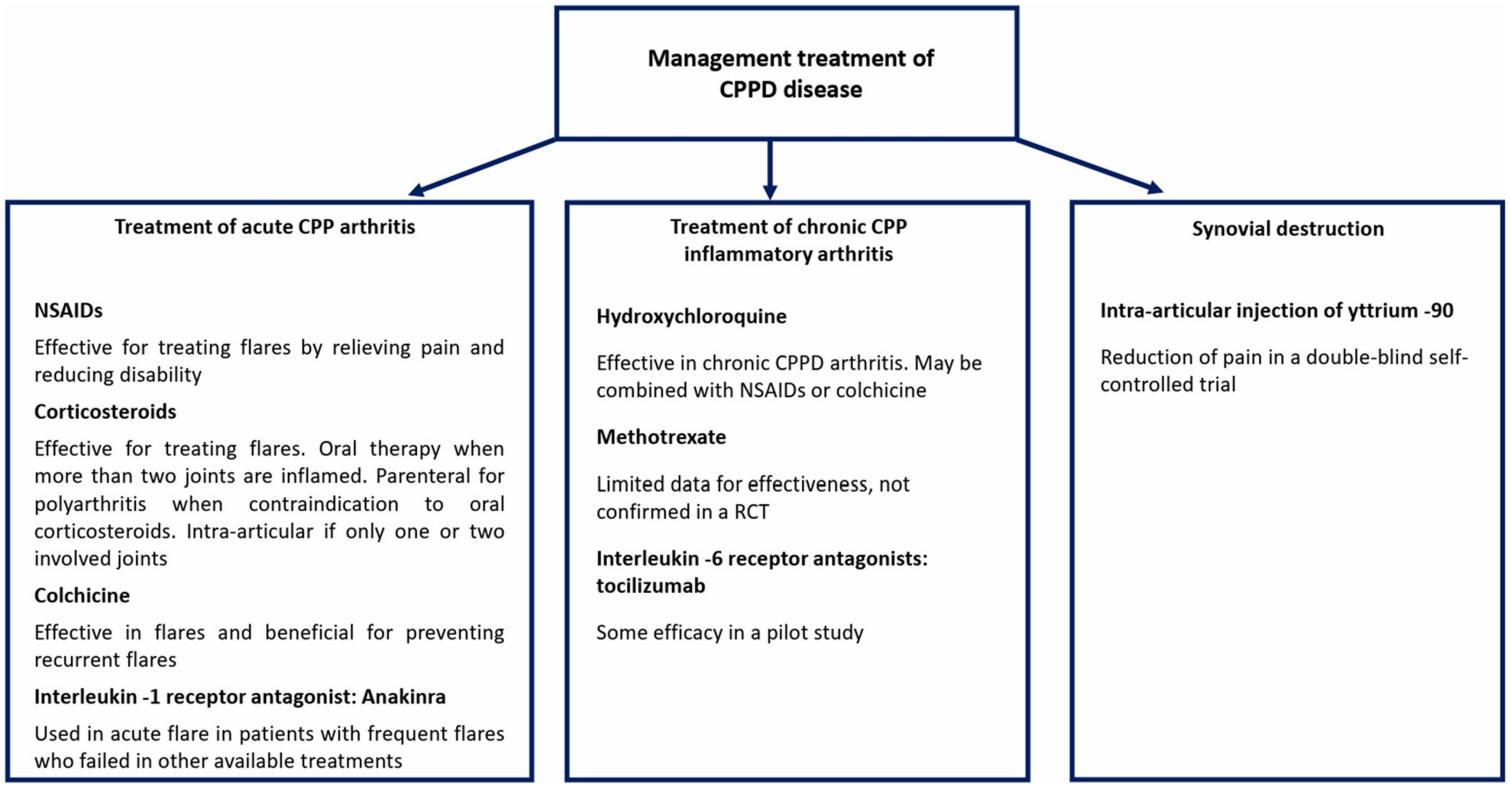
Management of acute and chronic CPPD arthritis and synovial destruction.

There are different treatment options for managing CPPD, including the potential use of immunosuppressants that may offer therapeutic benefits. Figure 2 summarizes the pharmacological management approaches currently available for CPPD disease.

### 4.1 Treatment of acute CPP arthritis

The treatment of an acute CPP crystal arthritis attack involves a combination of local and systemic measures (14–16). Non-pharmacological methods such as applying ice or cool packs and resting can alleviate pain and swelling temporarily. Symptomatic relief can be achieved through supportive measures such as restricting weight-bearing or routine joint use for 48–72 h and using splints when necessary (19). The choice of initial treatment depends on factors such as the number of affected joints, feasibility for joint injections, clinical features, patient’s medical history, and comorbidities (14). Intraarticular glucocorticoid injection, NSAIDs, oral or parenteral glucocorticoids, and colchicine are some of the treatment options available for acute CPP arthritis.

### 4.2 Initial treatment -one or two joints involved

For patients with acute CPP arthritis that affects one or two joints, joint aspiration and intraarticular glucocorticoid injection are suggested (14). If joint injection is not possible, oral anti-inflammatory agents are used instead. In a retrospective study, joint aspiration followed by intra-articular glucocorticoids has been shown to lead to faster resolution of synovitis than other treatment options (16). For large joints like knees and shoulders, triamcinolone acetonide (40–80 mg) mixed with 1 or 2 mL of lidocaine can be used, while smaller doses of glucocorticoid preparations can be used for smaller joints. Joint injection should only be performed once a septic joint has been ruled out. After injection, pain and swelling usually subside within 8–24 h. However, if symptoms worsen shortly after injection, fail to improve within 48 to 72 h, or if additional joints become inflamed, oral anti-inflammatory medication may be necessary.

### 4.3 Initial treatment—more than two joints

Systemic anti-inflammatory agents such as NSAIDs, colchicine, and glucocorticoids are indicated when more than two joints are involved or small joints are not suitable for intra-articular glucocorticoid injection (14, 16).

#### 4.3.1 NSAIDs

According to the CPPD EULAR guidelines, NSAIDs are considered as an effective treatment for acute CPP arthritis and prophylaxis, based on expert consensus opinion (14). Although RCTs have not evaluated their use in CPPD, they are widely used in gout treatment, and any NSAID can be used as there is no evidence to show differences in effectiveness (20). Traditional NSAIDs and selective COX-2 inhibitors are both viable options. Adverse effects, such as gastrointestinal (GI) intolerance and worsening of renal function, are uncommon with brief therapy courses (21). However, the use of NSAIDs in older patients with CPPD is often limited due to co-morbidities or contraindications, such as chronic kidney disease, active duodenal or gastric ulcer, heart failure, or difficult-to-control hypertension, NSAID allergy, and ongoing treatment with anticoagulants. When initiated during a flare, NSAID treatment provides pain relief and reduces disability. Following a significant improvement of symptoms, it is common practice to reduce the dosage while maintaining the frequency of dosing for a few days. NSAIDs may be discontinued 1–2 days after clinical signs resolve (20, 21).

#### 4.3.2 Colchicine

The EULAR strongly recommends the use of colchicine to manage acute CPP crystal arthritis, which has been supported by expert opinions (14). Colchicine works by disrupting microtubule polymerization, which reduces neutrophil chemotaxis and cell adhesion to achieve its anti-inflammatory effect. Though the exact mechanism is not entirely clear, studies have shown that it can reduce CPP and monosodium urate crystal-induced IL-1β expression *in vitro* (22).

A treatment option for acute CPP arthritis entails the initiation of low-dose colchicine regimens within 24 h of the onset of a flare, with no more than 1.5–1.8 mg on the first day, followed by 0.5 or 0.6 mg colchicine taken twice daily until the attack subsides (23). This option compares favorably with non-steroidal anti-inflammatory drugs or glucocorticoids and is less likely to result in rebound flares with treatment discontinuation after attack resolution than oral glucocorticoids. Studies conducted in 1980 indicated that intravenous colchicine is effective in improving pain, especially if administered within 24 h of attack onset (24). However, due to its association with a higher risk of adverse events, including death, it should be avoided (25, 26).

The most common adverse effects of colchicine are GI symptoms, such as diarrhea, abdominal pain, nausea, and vomiting. However, these symptoms are less likely to occur in patients who receive small doses of the drug (25). Reversible peripheral neuropathy is not a frequent occurrence during the brief period of colchicine administration. Severe toxicities such as cytopenias, rhabdomyolysis or myopathy, liver failure, or severe skin eruption are rare in patients receiving colchicine for short periods (23, 26, 27). The administration of colchicine is contraindicated when co-administered with a medication that strongly inhibits the cytochrome system component CYP3A4, or a medication that inhibits the membrane *P*-glycoprotein multidrug resistance transporter (*P*-gp) in the presence of renal or hepatic impairment (23). Clinicians must exercise caution and review patients’ medical history and concurrent medication usage before prescribing drugs like statins and colchicine together, as there is a significant risk of adverse events such as myopathies and rhabdomyolysis (27–29).

Studies have shown that oral colchicine may be effective in reducing the number of flares in CPPD patients (30, 31). A low dose of colchicine (0.6 mg BID) has been found to decrease the flare-up ratio from 3.2/patient/year to 1/patient/year (30), while in another study colchicine has been shown to reduce the annual attacks from 9.3 to 2.4 in 12 patients (31). Finally, a study of 39 patients with knee OA showed that adding colchicine 0.5 mg twice daily to the existing treatment plan of intra-articular glucocorticoids and oral piroxicam improved pain relief (32).

The COLCHICORT trial compared low-dose colchicine with oral prednisone in older patients with acute CPPD arthritis. Both treatments showed equivalent short-term efficacy in alleviating joint pain at 24 h but had different safety profiles. Colchicine was linked with a higher incidence of non-severe diarrhea, while prednisone was associated with hypertension and hyperglycemia (33).

#### 4.3.3 Corticosteroids

Although EULAR guidelines recommend the use of corticosteroids in treating CPPD, there are few studies available that evaluate their effectiveness (14). If patients are not suitable for NSAIDs and colchicine or if they cannot have intraarticular glucocorticoid injection, systemic glucocorticoids are used. Prednisone (or another equivalent oral glucocorticoid) is usually prescribed at a dose of 30–50 mg once daily or in two divided doses until there is an improvement in the flare. After that, the dose is tapered, which can take around 10–14 days. Usually, response to oral glucocorticoids is observed within 2 or 3 days, especially when one or two joints are affected, but it may take more time when there are many inflamed joints (14–16).

The administration of glucocorticoids requires careful consideration in patients suffering from heart failure, poorly controlled hypertension, or glucose intolerance. However, patients with moderate to severe renal insufficiency can safely receive glucocorticoids (34).

Parenteral glucocorticoids are used in doses equivalent to the suggested oral dose in patients who cannot take oral agents. A prospective cohort study evaluated the use of intramuscular triamcinolone for acute CPPD in 14 patients. All patients had a good clinical response, with at least 50% improvement in the patient and physician global assessments, while flare resolution occurred by day 4 in 13 out of 14 patients (35).

#### 4.3.4 Synthetic adrenocorticotropic hormone

The ACTH may serve as a viable alternative for acute CPP arthritis in patients who have contradictions to the use of corticosteroids, NSAIDs, and colchicine (14–16). Daoussis et al. (36) presented a case series of 14 patients with acute CPP arthritis treated with tetracosactide, an ACTH analog. They reported significant pain reduction within 24 h in 13 out of 14 patients. The remaining patient required a second dose the following day for complete resolution of arthritis. Another case series of five patients with acute CPP reported resolution of flares within an average of 4.2 days following administration of ACTH. However, it is important to note that one patient developed fluid overload and hypokalemia, indicating the need for careful monitoring of patients receiving ACTH therapy (37).

#### 4.3.5 Anakinra

Anakinra is an IL-1 receptor antagonist (IL-1Ra) that is administered as a daily 100 mg subcutaneous injection. Studies have demonstrated that CPP crystals induce the downregulation of the natural IL-1Ra antagonist, resulting in increased IL-1 activity. This leads to a rise in cytokines such as tumor necrosis factor-alpha (TNF-α), IL-6, and chemokines (38, 39).

Anakinra has been utilized in patients with frequent flares who have failed to respond to other available treatments or in those who experience “rebound flares” despite appropriate tapering of glucocorticoid treatment (40). Typically, a 3-day regimen has been used to treat acute attacks. However, patients with frequently recurrent or persistent arthritis may also benefit from daily or every other day doses as a maintenance therapy (41).

Anakinra has shown efficacy in CPP crystal arthritis based on case reports and cohort studies (42–51). McGonagle et al. (42) reported the first case of using anakinra for steroid-resistant CPPD. After treatment with anakinra, the patient showed improvement in 14 days and was symptom-free after 3 months.

Anakinra has also showed a prompt clinical response in several retrospective cohort studies. In a study of five patients, four showed a mean time of 3 days from symptom onset to recovery post-treatment (45). However, one patient did not respond to treatment. Other studies showed efficacy in 79–87.5% of patients (47, 49). During follow-up periods, relapses occurred in 37.5% of patients, with a mean time to relapse of 3.4 months (47). In the largest retrospective cohort study, with 33 patients on day four of treatment with anakinra, there was a significant reduction in the mean tender joint count (from 5.8 to 1.0), swollen joint count (from 3.9 to 0.9), VAS pain score (from 64.8 mm to 21.2 mm), and C-reactive protein (CRP) (from 116.1 to 26.0 mg/L). A good clinical response was observed in 81.8% of patients, while five patients had no or partial response. However, nine patients experienced a relapse with a mean time to relapse of 2.1 months (50). One case of injection-site reaction (45), one case of rash (49), and one case of bacterial pneumonia (50) were adverse effects associated with anakinra administration.

A systematic literature review by Cipolletta et al. (40) included 74 patients with CPPD disease who received anakinra. Anakinra was primarily used in cases of refractory disease (85.1%) or when patients had contraindications to standard treatments (23.0%). Anakinra demonstrated a clinical response in 80.6% of patients with acute CPP arthritis, significantly reducing total joint count, swollen joint count, VAS pain, and CRP (40). Adverse events were reported in 4.1% of patients, with skin reactions and respiratory infections being the most common.

A recent study by Dumusc et al. (52) compared anakinra and prednisone for treating acute CPPD in a controlled, double-blinded RCT of 15 patients. The study found that anakinra had a faster onset of action than prednisone and could be a better choice for patients with comorbidities (52). Still, direct studies comparing the cost-effectiveness of anakinra versus corticosteroids in acute CPPD are lacking (53). Thus, physicians should base treatment decisions on clinical judgment, patient-specific factors, and available evidence regarding the safety and efficacy of anakinra and corticosteroids for acute CPPD.

### 4.4 Treatment of resistant acute CPP arthritis

Most acute flares resolve within 7–14 days, especially if the patient is treated early in the attack (1, 2). Thus, genuinely resistant disease is uncommon, although some attacks may resolve slowly, particularly if treatment is not started early or prior flares have led to chronic arthropathy with almost continuous joint inflammation. Nevertheless, if symptoms are not improving as expected, the patient’s adherence to the treatment course should be assessed, alternative agents should be considered, and diagnosis should be reevaluated, excluding other causes of acute arthritis such as infection (14–16). The management of patients with persistent symptoms of a confirmed acute flare depends on the prior therapy and the existence of comorbidities.

Thus, in some patients being treated with NSAIDs a more prolonged than usual course of therapy may be required. Patients resistant to an adequate course of NSAIDs may respond to glucocorticoids. Patients treated with colchicine without improvement benefit from switching to NSAIDs or glucocorticoids. Recurrent (or “rebound”) flares treated with glucocorticoids may require slower tapering of the dose with an extension of therapy (10–21 days) (14–16). In patients with refractory acute CPP, arthritis may be treated with anakinra (45).

### 4.5 Prophylaxis of acute CPP crystal arthritis

For patients who experience three or more attacks, it is suggested to take colchicine (0.5 or 0.6 mg twice daily) as a prophylaxis (14, 30). Some patients may find it more tolerable to reduce the dose to 0.5 or 0.6 mg once daily or every other day, although there is no evidence to support the reduction of flares with lower doses.

If colchicine alone does not provide an adequate response, a lower dose of NSAIDs may be used instead of or in addition to colchicine therapy.

### 4.6 Treatment of chronic CPP crystal arthritis

A subset of patients with CPP arthritis may present with symptoms of chronic joint inflammation that can be mistaken for RA. In such cases, prolonged administration of the lowest possible dose of NSAID may be effective in controlling symptoms. However, it is important to consider the patient’s age, comorbidities, and concomitant medication use (14–16). Alternative therapeutic options include using colchicine (0.5 or 0.6 mg twice daily) for 8–12 weeks or low-dose oral glucocorticoids, such as prednisone, in doses not exceeding 7.5–10 mg daily (41).

#### 4.6.1 Hydroxychloroquine

Hydroxychloroquine (HCQ) has been evaluated in CPPD disease in one double-blind, randomized crossover study of 36 patients (19 treated with HCQ vs. 17 with placebo) (54). The dosage of HCQ was started at 100 mg/day and was increased every month to a maximum of 400 mg/day for non-responders. A response rate (as defined by at least a 30% reduction in the number of swollen and tender joints) was seen in 76% of the treatment group compared with 32% in the placebo group. During the open-label period of the study, 85% of patients who were given the placebo and then crossed over to HCQ showed treatment responses. The study population was not clearly defined, and non-validated outcome measures were used. However, EULAR recommendations include HCQ for chronic inflammatory arthritis with CPPD (14–16).

#### 4.6.2 Methotrexate

Methotrexate (MTX) has been investigated as a potential treatment for CPPD in several studies (55–57). In a case series of five patients, MTX resulted in a significant decrease in VAS pain (*p* < 0.001), number of swollen and tender joints (*p* < 0.001) and a decrease in erythrocyte sedimentation rate and CRP. The mean time to symptom improvement was 7.4 weeks (range 4–16 weeks) (55). Another retrospective cohort study with 10 patients exhibiting refractory CPP arthritis reported a positive response to MTX, as evidenced by a median VAS pain reduction of 74 mm (56). Based on these positive outcomes, EULAR recommended MTX as a treatment for severe refractory CPPD (14–16).

However, a double-blind, randomized crossover trial involving 26 patients with recurrent or persistent chronic CPP arthritis presented contrasting results (57). The study found no significant change in disease activity scores, number of tender or swollen joints, CRP levels, analgesic pill usage, number of flares in 3 months, or VAS pain scores between MTX and placebo. Several methodological issues may have impacted the study’s findings (58). These issues include the trial’s limited sample size, the utilization of the DAS 44 as an outcome measure in CPPD without validation, and the inclusion of different CPPD phenotypes, such as acute and chronic, mono, oligo, and polyarticular. As such, the authors suggest the need for more extensive and prolonged studies with well-defined cohorts and validated outcome measures to accurately assess MTX’s efficacy in CPPD disease (59).

#### 4.6.3 Biologic agents

##### 4.6.3.1 Anakinra

Cipolletta et al. (40) reported that in chronic CPP arthritis, the response rate to anakinra was 42.9%. However, the small sample size did not allow for a definitive conclusion. Nevertheless, all the studies included in the analysis showed that anakinra could prevent the onset of new flares in chronic CPP arthritis.

##### 4.6.3.2 Anti-TNFα agents

The utilization of TNFα blockers in CPPD is restricted. So far, there are only three patients with chronic CPP inflammatory arthritis treated with anti-TNFα agents, reporting conflicting results. Efficacy of anti-TNF agents was reported in two patients treated with infliximab, whereas no response was observed with adalimumab. Infliximab was maintained for 9 years without loss of efficacy and serious adverse events. A case of recurrent attacks of CPPD in a patient with RA treated with etanercept (60) implied that different inflammatory pathways in CPPD and RA are involved. It seems that in CPPD there is a minor role of TNFα blockers and a more important role of IL-1 inhibitors in its pathogenesis.

##### 4.6.3.3 Tocilizumab

The use of tocilizumab, a monoclonal antibody targeting the IL-6 receptor, has been tested in treating persistent CPP arthritis (61, 62). An open-label pilot study involving 11 CPPD patients with either prior treatment failure, contraindication, or intolerance to other medications, showed promising results with tocilizumab (62). After 3 months, the median global assessment VAS significantly reduced from 60 to 15 (*p* = 0.006), and treatment efficacy was sustained during a median 10-month follow-up. Notably, some patients were able to taper or discontinue prednisone. However, adverse effects were observed, including dyspnea attributed to tocilizumab and a lung abscess requiring hospitalization, surgical drainage, and antibiotics. Despite these adverse events, the overall findings suggest that tocilizumab holds promise in CPPD treatment and warrants further evaluation. The potential risk of infections, especially in older individuals, should be carefully considered in the management of chronic CPPD disease.

### 4.7 Other therapies

#### 4.7.1 Glycosaminoglycan polysulfate

A prospective self-controlled trial has been conducted to assess the efficacy of intraarticular glycosaminoglycan polysulfate (GAGP) in 12 patients with CPPD (63). All patients presented with bilateral disease, and the GAGP was administered into the most severely affected joint, while the contralateral joint served as a control. The trial demonstrated a significant reduction in pain (*p* < 0.01) and improvement in joint mobility (*p* < 0.001) that persisted for a 1-year follow-up period. During the follow-up period, 4 treated and 9 control joints exhibited acute arthritis.

#### 4.7.2 Hyaluronic acid

The efficacy of intraarticular hyaluronic acid in patients with CPPD remains a topic of debate. A case report documented recurrent acute CPP arthritis following hyaluronic acid injection (64) and three other cases reported CPP arthritis triggered by hyaluronic injection (65, 66). Conversely, a case series of eight patients demonstrated significant improvement in joint mobility and severe pain after intraarticular injection of hyaluronic acid for chronic CPPD (67). Notably, hyaluronic acid may be administered to patients with coexistent OA and CPPD (68). These findings suggest that the use of intraarticular hyaluronic acid in patients with CPPD should be approached with caution, and that further research is needed to determine its safety and efficacy.

#### 4.7.3 Synovial destruction

Two studies have evaluated the destruction of the synovial membrane in patients with CPPD (69, 70). In the first study, which was a double-blind self-controlled study of 15 patients, radiation synovectomy was performed by injecting intraarticular yttrium-90 (5 mCi) plus triamcinolone hexacetonide 20 mg into one knee and saline plus triamcinolone into the other knee as a control. After 6 months, a statistically significant reduction in pain, inactivity, stiffness, and effusion was observed in the treated knees compared to the controls (*p* < 0.05) (69).

In the second study, 49 patients were evaluated for the effectiveness of laser irradiation. Among them, 26 patients were designated for the treatment group, while the remaining patients were assigned to the comparison group, where they received diclofenac. The response rate was 69.2% for the treatment group and 60.8% for the comparison group (*p* < 0.05) (70). Although the authors reported positive results, no other studies have been conducted to confirm these findings.

#### 4.7.4 Magnesium supplementation

The administration of magnesium supplements has been proposed as a potential treatment for CPPD, regardless of the initial magnesium levels. A double-blind, randomized, placebo-controlled trial was conducted on 38 patients, with 19 of them receiving 30 mEq of magnesium carbonate daily for a period of 6 months (71). The results of the study indicated a significant improvement in pain score, joint swelling, and tenderness in the treatment group, although a pronounced placebo effect was also observed. Therefore, further investigation is warranted to determine the precise role of magnesium in the treatment of CPPD.

## 5 Summary

In conclusion, the management of CPPD poses unique challenges due to the limited number of RCTs specific to the disorder. Treatments borrowed from gout management can provide symptomatic relief and target acute flares, but it is also important to address underlying disorders associated with CPPD. The approach to acute CPP arthritis involves interventions such as triamcinolone injection and oral anti-inflammatory therapy based on individual patient assessments. Colchicine prophylaxis is advised for recurrent flares, while chronic CPP inflammatory arthritis may necessitate a combination of drugs such as NSAIDs, colchicine, hydroxychloroquine, glucocorticoids, and or methotrexate.

In cases of treatment resistance or refractory flares, biological therapies like anakinra and tocilizumab may be considered. However, it is important to note that, as of now, no disease-modifying therapies effectively reduce articular calcification in CPPD. Managing CPPD necessitates a detailed and tailored approach that integrates individualized patient assessments, medical history, comorbidities, and response to treatment. Further research and clinical trials are warranted to enhance our understanding of CPPD and refine treatment strategies for this condition.

## Author contributions

PV: Writing – original draft. AV: Data curation, Writing – original draft. AD: Writing – review & editing.
